# Comparative Effectiveness of Radiofrequency or Microwave Ablation and Chemotherapy for the Treatment of Large (>5 cm) Hepatocellular Carcinoma Tumors

**DOI:** 10.7759/cureus.106552

**Published:** 2026-04-06

**Authors:** Artem Boyev, Nirav Thosani, Putao Cen, Jennifer M Bailey-Lundberg, Julie Rowe, Curtis J Wray

**Affiliations:** 1 Department of Surgery, University of Texas Health Science Center at Houston McGovern Medical School, Houston, USA; 2 Center for Interventional Gastroenterology at UTHealth (iGUT), University of Texas Health Science Center at Houston McGovern Medical School, Houston, USA; 3 Department of Internal Medicine, Division of Oncology, University of Texas Health Science Center at Houston McGovern Medical School, Houston, USA; 4 Department of Anesthesiology, University of Texas Health Science Center at Houston McGovern Medical School, Houston, USA

**Keywords:** advanced hepatocellular carcinoma, cancer immunotherapy, hepatocellular carcinoma, liver neoplasm, local therapy, percutaneous ablation, percutaneous microwave ablation, primary liver neoplasm, radio-frequency ablation, thermal ablation

## Abstract

Background and objectives

Treatment of hepatocellular carcinoma (HCC) >5 cm is problematic, as patients are often outside transplantation criteria, and cirrhosis compromises curative resection. The purpose of this study was to evaluate survival following treatment with ablation compared to chemotherapy in large HCC.

Methods

This was a retrospective cohort study of the National Cancer Database (NCDB) Participant User File from 2004 to 2017. Patients with HCC >5 cm in diameter treated with ablation versus chemotherapy alone were compared. Inverse probability-weighted propensity scores were used to model treatment assignments in the Cox proportional hazards model.

Results

A total of 14,783 HCC patients with tumors >5 cm and <9 cm were treated with chemotherapy (N=14,127) or ablation (n=656). After adjustment for stage, comorbidity, insurance status, facility type, and race, survival was significantly improved with ablation. The treatment effect of ablation was estimated using inverse-probability-weighted propensity scores, and the survival advantage was 19 months longer (p<0.001; 95% CI: 10.1-28.2) compared to chemotherapy.

Conclusions

Despite the reluctance to use ablation in lesions >5 cm, our analysis suggests a survival advantage when compared to chemotherapy. These results are promising, and future trials should evaluate ablation in combination with other local and systemic therapies.

## Introduction

Liver cancer is the sixth most deadly cancer in the United States, with over 35,000 new diagnoses and 28,000 deaths per year [1,2]. The incidence of liver cancer increased from 4.8 per 100,000 people in 1999 to 8.4 per 100,000 people a decade later in 2019, and liver and bile duct cancer was expected to affect 41,260 new patients in 2022 [1,3-5]. Hepatocellular carcinoma (HCC) is responsible for approximately 90% of all primary liver cancers [6]. Patients with chronic liver disease experience hepatic inflammation progressing to dysplasia, culminating in HCC following additional molecular alterations [4]. Over 90% of HCC occurs following chronic liver disease, and cirrhosis is the strongest risk factor [6]. As a result of the disease course and biology, HCC patients often have liver disease and cirrhosis that significantly complicate management.

Treatment decisions are frequently based on the Barcelona-Clinic Liver Cancer (BCLC) staging classification, and treatment recommendations consider tumor size, degree of liver disease, and functional status [7]. Early tumors with limited involvement are curable with resection; however, underlying cirrhosis and portal hypertension often make resection unfeasible. Liver transplantation is another treatment option for limited tumors, but tumors >5 cm are outside of the Milan transplantation criteria [4,8]. As patients with HCC >5 cm in the setting of moderate or advanced liver disease are likely not candidates for resection or transplantation, they have limited treatment options outside of systemic therapy.

Radiofrequency or microwave ablation is traditionally used for smaller tumors in patients who are not candidates for surgery or transplantation [4,5,9]. Ablation is usually avoided in tumors larger than 3-4 cm in diameter because the extent of tumor necrosis and the likelihood of achieving a negative margin are thought to decrease in larger tumors [4,5,9]. There is also a risk of thermal damage to nearby bile ducts or vasculature and a heat sink effect when tumors are near large vessels [9]. Despite these limitations, it is unclear if ablation can offer patients with large (>5 cm) HCC a survival advantage compared to systemic therapy. The goal of the current study was to investigate survival associated with radiofrequency or microwave ablation compared to systemic chemotherapy in large (>5 cm) HCC lesions. We hypothesized that ablation would be associated with improvements in the primary outcome of overall survival compared to chemotherapy. This article was previously presented as a poster presentation at the 2022 Society of Surgical Oncology (SSO) Annual Meeting on March 11, 2022.

## Materials and methods

In this retrospective cohort study, we reviewed the National Cancer Database (NCDB) Participant User File for HCC for the available dates of 2004 to 2017. Exclusion criteria were resection or transplantation, ablation method other than radiofrequency or microwave (including ethanol or cryotherapy ablation), largest lesion less than 5 cm or greater than 9 cm, and missing vital status. The NCDB file does not differentiate between conventional chemotherapy and targeted therapy, so all systemic anticancer therapies were categorized as “chemotherapy.” Radiofrequency and microwave ablation were both included in the ablation group. Comorbidity was graded on the Charlson-Deyo 0-5 scale [10,11]. Scores 3-5 were combined into one category for analysis. Staging was according to the American Joint Committee on Cancer (AJCC) TNM staging system [12]. Overall survival was the primary outcome measure. 

Descriptive statistics were used to quantify demographic, clinico-oncologic, and postoperative variables. Pearson's chi-square was used to compare groups. An alpha level of 0.05 was set, and all tests were two-sided. A Cox proportional hazards model was created for the primary outcome of all-cause mortality. A subset analysis was performed using only patients with Stage I and II disease. Multivariable regression-adjusted propensity score models were created to compare predicted survival duration for patients who received ablation versus chemotherapy. A second model was created to compare predicted survival duration for ablation plus chemotherapy versus only chemotherapy. A third model was created to compare ablation versus chemotherapy adjusted for stage.

To minimize confounding related to nonrandom treatment selection (ablation versus chemotherapy), inverse probability of treatment weighting based on propensity scores was utilized. Propensity scores were estimated using multivariable logistic regression to model the probability of receiving the treatment of interest as a function of baseline patient, tumor, and facility characteristics selected a priori based on clinical relevance and existing literature.

Stabilized inverse probability weights were applied to improve precision and reduce the influence of extreme values. Adequate overlap of propensity score distributions between treatment groups was assessed visually to confirm common support. Balance of baseline covariates after weighting was evaluated using standardized mean differences.

Treatment effects were estimated using weighted regression models in the resulting pseudo-population. For time-to-event outcomes, weighted Cox proportional hazards models with robust variance estimation were used to generate hazard ratios (HRs) and corresponding 95% CIs. The proportional hazards assumption was assessed using Schoenfeld residuals.

Sensitivity analyses included truncation of extreme weights and comparisons with conventional multivariable regression models to assess the robustness of findings. All statistical analyses were performed using Stata 16 (StataCorp, College Station, TX, USA). The study was exempt from Institutional Review Board (IRB) review.

## Results

There were 14,783 patients with HCC between 5 and 9 cm (Table 1). Chemotherapy was administered to 95.6% (n=14,127), and ablation was performed in 4.4% (n=656). Treatment choice differed significantly by race; ablations were less frequently performed in African American patients and more frequently performed in Hispanic patients (p=0.010). Treatment choice differed significantly between insurance status (p<0.001) and median income (p=0.034). Patients treated with ablation had slightly higher comorbidity scores (p < 0.001). Ablation patients had less advanced disease. Stage I patients made up 40.4% of those treated with ablation compared with only 20.0% of those treated with chemotherapy (p < 0.001). In contrast, almost 60% of those treated with chemotherapy had Stage III or Stage IV disease, compared with about 30% of those treated with ablation.

**Table 1:** Demographics, comorbidities, stage at presentation, and readmissions by treatment type Values are listed as count and percentage of the column unless otherwise specified.

		Chemotherapy	Ablation	Pearson's chi-square
Total (% row)	n=14,783	14,127 (95.6%)	656 (4.4%)	
Sex (n=14,783)	Male	10,810 (76.5%)	505 (77.0%)	p=0.785
	Female	3,317 (23.5%)	152 (23.0%)	
Race (n=14,783)	Caucasian	8,394 (59.4%)	395 (60.2%)	p=0.010
	Black/African American	2,164 (15.3%)	72 (11.0%)	
	Hispanic	2,357 (16.7%)	129 (19.7%)	
	Asian/Other	1,212 (8.6%)	60 (9.1%)	
Insurance (n=14,783)	Not insured	737 (5.2%)	16 (2.4%)	p<0.001
	Private	4,464 (31.6%)	181 (27.6%)	
	Medicaid	1,933 (13.7%)	80 (12.2%)	
	Medicare	6,496 (46.0%)	357 (54.4%)	
	Other government	257 (1.8%)	13 (2.0%)	
	Unknown	240 (1.7%)	9 (1.4%)	
Median income (n=14,210; USD$)	<38,000	3,294 (24.3%)	138 (21.6%)	p=0.034
	38,000-47,999	3,266 (24.1%)	136 (21.3%)	
	48,000-62,999	3,569 (26.3%)	197 (30.9%)	
	63,000+	3,443 (25.4%)	167 (26.2%)	
Charlson-Deyo Score (n=14,783)	0	8,118 (57.5%)	320 (48.8%)	p<0.001
	1	3,290 (23.3%)	170 (25.9%)	
	2	1,082 (7.7%)	68 (10.4%)	
	3+	1,637 (11.6%)	98 (14.9%)	
Stage (n=14,783)	Stage 0	1 (0.0%)	0 (0.0%)	p<0.001
	Stage I	2,828 (20.0%)	265 (40.4%)	
	Stage II	1,056 (7.5%)	87 (13.3%)	
	Stage III	5,658 (40.1%)	170 (25.9%)	
	Stage IV	2,728 (19.3%)	26 (4.0%)	
	Not applied	602 (4.3%)	10 (1.5%)	
	Unknown	1,254 (8.9%)	98 (14.9%)	
Readmission within 30 days (n=14,783)	0	13,866 (98.2%)	595 (90.1%)	p<0.001
	1	76 (0.5%)	32 (4.9%)	
	2	39 (0.3%)	10 (1.5%)	
	3	3 (0.0%)	0 (0.0%)	
	Missing	143 (1.0%)	19 (2.9%)	

On univariate analysis including all stages of disease, treatment with ablation was associated with a 40% increase in likelihood of survival compared to chemotherapy alone (HR 0.60, p <0.001, 95% CI 0.49 - 0.73; Table 2). Among patients with Stage I or II disease, the increased likelihood of survival was unchanged (HR 0.59, p<0.001, 95% CI 0.45 - 0.77). When compared with Caucasian patients, African American patients (HR 1.27, p<0.001, 95% CI 1.11 - 1.45) and Hispanic patients (HR 1.23, p=0.001, 95% CI 1.09 - 1.39) had a higher likelihood of all-cause mortality. When compared with patients who were uninsured, patients who carried insurance had a lower likelihood of all-cause mortality irrespective of the type of insurance (all p<0.05). When compared with a score of 0, a comorbidity score of 1 or 2 did not significantly affect the likelihood of mortality. However, a score of 3 or more was significantly associated with increased mortality (HR 1.18, p=0.036, 95% CI 1.01 - 1.38).

**Table 2:** Univariate Cox proportional hazards model of predictor variables and overall survival adjusted for race, insurance status, and comorbidity. Abbreviations: HR, hazard ratio; CI, confidence interval

		HR	P-value	95% CI
Treatment	Ablation (all Stages)	0.60	<0.001	0.49 - 0.73
	Ablation (Stage I/II)	0.59	<0.001	0.45 - 0.77
Age	Age	1.01	0.061	1.00 - 1.01
Race (vs. Caucasian)	Black/African American	1.27	<0.001	1.11 - 1.45
	Hispanic	1.23	0.001	1.09 - 1.39
	Asian	1.02	0.774	0.88 - 1.19
Insurance (vs. Uninsured)	Private Insurance	0.57	<0.001	0.47 - 0.69
	Medicaid	0.74	0.006	0.60 - 0.92
	Medicare	0.54	<0.001	0.44 - 0.66
	Other government	0.66	0.021	0.47 - 0.94
	Unknown	0.86	0.430	0.59 - 1.26
Charlson-Deyo Comorbidity Index (CCDC vs. Score 0)	CDCC Score 1	0.99	0.852	0.88 - 1.11
	CDCC Score 2	0.98	0.804	0.82 - 1.16
	CDCC Score 3 or more	1.18	0.036	1.01 - 1.38

**Figure 1:**
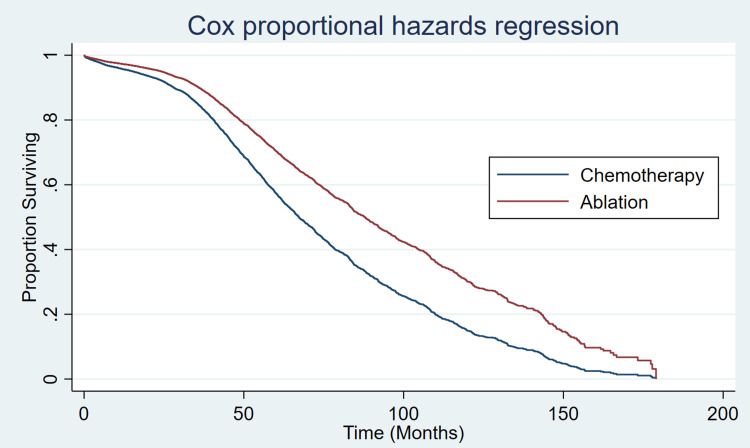
Cox proportional hazards regression model of predicted survival (in months) for treatment with ablation versus chemotherapy

To further examine the specific treatment effect of ablation, regression-adjusted propensity score modeling adjusted for race, insurance status, facility type, and comorbidity showed ablation was associated with an 18.8-month increase in survival (p<0.001, 95% CI 9.67 - 28.02; Table 3, Figure 1). Given the difference in cancer stage between the chemotherapy and ablation groups, an additional model was created to include cancer stage. Compared with chemotherapy, ablation was associated with a 19.1-month survival increase (p<0.001, 95% CI 10.07 - 28.22) when adjusted for stage, race, insurance status, facility type, and comorbidity. Although not the specific focus of this project, the combination of ablation and chemotherapy compared to ablation alone was examined. When adjusted for other factors, the treatment with both ablation and chemotherapy (n=476 patients) was associated with a 13.1-month (p= 0.005, 95% CI 3.90 - 22.37) increase in survival compared to chemotherapy alone.

**Table 3:** Regression-adjusted propensity score model of survival by treatment type Models 1 and 2 adjust for comorbidity, insurance status, facility type, and race. Model 3 adjusts for stage, comorbidity, insurance status, facility type, and race.

		Survival increase	p-value	95% CI
Model 1	Ablation (vs. Chemotherapy)	18.8 months	<0.001	9.67 - 28.02 months
Model 2	Ablation (vs. Chemotherapy)	19.7 months	<0.001	10.31 - 29.01 months
	Ablation + Chemotherapy (vs. Chemotherapy)	13.1 months	0.005	3.90 - 22.37 months
Model 3	Ablation (vs. Chemotherapy)	19.1 months	<0.001	10.07 - 28.22 months

## Discussion

This was a retrospective cohort study of a large NCDB data set of nearly 15,000 patients with large (>5 cm) HCC who were treated with ablation or chemotherapy alone. While only 4% of patients were treated with ablation, this study nevertheless highlights how ablation has been used in certain cases of large (>5 cm) HCC. When adjusted for race, insurance status, facility type, comorbidity, and stage, patients treated with ablation had longer survival compared to chemotherapy alone. These findings are intriguing and suggest ablation may have a role in the treatment of patients with large (>5 cm) HCC who are not candidates for resection or transplantation, and that this treatment modality may provide a survival advantage compared to systemic chemotherapy.

Radiofrequency or microwave ablation is a potentially curative therapy for single tumors <2 cm or multiple tumors in patients who are poor surgical candidates [9]. Used as a first-line therapy for early HCC, radiofrequency ablation has resulted in a complete response rate of 70%-90% and a median overall survival of 60 months, albeit with high five-year recurrence rates of 50%-70% [9]. While the current study did not separate microwave ablation from radiofrequency ablation, the results of both techniques have been comparable [13]. A randomized controlled trial of 152 patients at European university hospitals comparing microwave ablation to radiofrequency ablation did not observe a difference in local tumor progression at two years, with 6% and 12% (p=0.27) showing progression [14]. Some recent studies have evaluated improved stereotactic ablation using 3D navigation for planning of multiple ablation zones to increase the possible ablation zone in larger tumors [15]. One retrospective study of this method found a complete response in 97.3% of treated lesions, to include 96.3% of lesions ≥ 3 cm and up to 8 cm, with no correlation between residual tumor and tumor size [15].

The patients in the current study all had at least one large HCC tumor between 5 and 9 cm and were not treated with resection or transplantation, presumably because they were outside of transplantation criteria and their tumors or underlying liver disease precluded surgical resection. These patients would traditionally not be considered for ablation, but ablation for larger HCC tumors has been explored with some success. In a retrospective study of 124 patients with large HCC, Jung et al. found median survival was 84.2 months for surgical resection and 74.1 months for ablation +/- transarterial chemoembolization, demonstrating that ablation was possible with comparable outcomes to surgical resection in large tumors [16]. Iezzi et al. performed a prospective single-center pilot study evaluating the efficacy of ablation combined with transarterial chemoembolization and drug-eluting beads [17]. In their group of 17 patients with HCC >5 cm, 70% of patients had an initial complete response, and 53% of patients had a sustained complete response after a mean follow-up of 23 months [17]. Like other studies of tumor arterial occlusion, Iezzi et al. used balloon occlusion of the arterial supply to decrease heat loss and increase the area of necrosis during the ablation of larger tumors [17,18]. In a small retrospective study of ablation versus resection, a subset of patients with HCC >5 cm who were treated with ablation demonstrated a median survival of 63 months that was similar to resection [19]. Other studies have found conflicting results; a retrospective, propensity-matched study found that survival was worse in patients with HCC tumors >5 cm who underwent ablation compared with resection, although median survival was still 21.5 months in the ablated group [20].

Contrary to traditional practice, there are studies that demonstrate the feasibility of ablation in large HCC tumors, but the benefits of local treatment by ablation may extend beyond local tissue destruction. By inducing necrosis in a central zone and heat stress in a peripheral zone, ablative therapies contribute to tumor antigen presentation [21]. Studies of radiofrequency ablation have demonstrated that T-cells have increased reactivity to autologous tumor antigens and hepatocellular cell lines [22]. Another study found that a higher number of tumor-specific T-cells was significantly correlated with the length of recurrence-free survival and was, in fact, the only prognostic factor associated with decreased recurrence [23]. Treatment with radiofrequency ablation may also increase the frequency, number, and expression of activating receptors in the natural killer cell population [24]. Treatment with microwave ablation has shown comparable results, as tumor-specific responses have been observed to increase following microwave ablation, and an increased response was associated with longer progression-free survival [25]. However, the tumor-specific response is only observed in 10%-50% of patients, and the response appears to wane after 24 weeks, suggesting the immune response following ablation may not be robust enough to completely prevent recurrence or eliminate metastatic deposits [21-25].

To address the above, combinations of immunotherapy with ablation have been evaluated to determine if systemic immunotherapy can capitalize on the anti-tumor response from local treatment [9,21]. Duffy et al. evaluated an immunotherapeutic with ablation in treatment-resistant HCC and observed a 12-month progression-free survival of 31%, alongside some patients who had a tumor response outside of the ablated area [26]. Given the efficacy of the immunotherapeutic atezolizumab in combination with bevacizumab in unresectable HCC, the combination of ablation and immunotherapy is an intriguing direction to investigate [27,28].

There are limitations to using the NCDB data set. Clinical details such as tumor number, tumor imaging, liver function parameters, Child-Pugh class, or performance status were not available in the NCDB dataset; all of these would have provided a more robust comparative model. Selection bias was likely present, as relatively few patients were treated with ablation, and those often had less advanced disease and likely more biologically favorable tumors. However, the survival benefit of ablation persisted when the analysis was restricted to Stage I-II disease and to models adjusted for stage. It was not possible to subdivide patients by the type of chemotherapy (single-agent targeted therapy versus combination chemotherapy) or by the type of ablation, and most patients likely received sorafenib, which is no longer standard of care [27-30]. It was difficult to determine the sequence of treatments and whether patients who received multiple modalities did so because of poor response or a long period of stable disease. Nevertheless, this is a study of a large national data set that demonstrates ablation is feasible and may offer a survival advantage compared to chemotherapy in some patients with large tumors.

## Conclusions

In this exploratory analysis of the NCDB, when adjusted for other factors, ablation was associated with longer overall survival compared to systemic therapy in patients with large HCC. Patients with HCC >5 cm have often exhausted surgical options, and ablation can offer local therapy that is potentially associated with longer overall survival compared to systemic therapy alone, especially with newer methods for delivering treatment and in combination with other modalities. Although incomplete ablation of larger lesions is possible, systemic, immune-mediated anti-tumor responses may complement treatment. Future trials should examine ablation in concert with newer modalities like immunotherapy in the treatment of large HCC.
